# DNA Type Influence on Properties of Thin Layer of DNA Complexes

**DOI:** 10.3390/ma18133022

**Published:** 2025-06-26

**Authors:** Aleksandra Radko, Katarzyna Makyła-Juzak, Robert Ekiert, Julia Chudzik, Dagmara Sokołowska, Sebastian Lalik, Monika Marzec

**Affiliations:** 1 Institute of Physics, Jagiellonian University, Łojasiewicza 11, 30-348 Kraków, Poland; aleksandra.radko@wp.pl (A.R.); julia.chudzik@doctoral.uj.edu.pl (J.C.); dagmara.sokolowska@uj.edu.pl (D.S.); sebastian.lalik@uj.edu.pl (S.L.); 2 Faculty of Chemistry, Jagiellonian University, Gronostajowa 2, 30-387 Kraków, Poland; katarzyna.makyla@uj.edu.pl; 3 Faculty of Biochemistry, Biophysics and Biotechnology, Jagiellonian University, Gronostajowa 7, 30-387 Kraków, Poland; robert.ekiert@uj.edu.pl; 4 Doctoral School of Exact and Natural Sciences, Jagiellonian University, Łojasiewicza 11, 30-348 Kraków, Poland

**Keywords:** DNA complex, thin layer, LB technique, AFM

## Abstract

In the search for new functional materials, strong emphasis is placed on the ecological aspect, which is why thin layers of materials based on deoxyribonucleic acid (DNA) are fascinating from the point of view of applications. Thin layers of DNA–cationic surfactant complexes were created on mica slices using the Langmuir–Blodgett deposition technique. Three cationic surfactants (CTMA, BAC, HDP) and two types of DNA (linear dsDNA and plasmid pDNA) were used to synthesise the complexes. It was shown that the pattern of the obtained layer depended on the lifting conditions, type of DNA, and type of surfactant. The elongated structures that formed along the layer lifting direction were examined by AFM imaging and fast Fourier transform analysis. The main difference between the layers formed by plasmid pDNA-based and linear dsDNA-based complexes was the thickness of the stripes and the minimum surface pressures at which elongated structures were formed.

## 1. Introduction

The deoxyribonucleic acid (DNA) has been extensively studied since the discovery of its structure in the mid-20th century, with a focus on its applications, initially in biology and biophysics, and, more recently, for its potential applications in technology. Over the last two decades, particular attention has been paid to DNA as a biopolymer widely available in nature, making it an economical and environmentally friendly alternative to artificial materials used in the rapidly growing fields of photonics [1,2], electronics [3,4], optoelectronics [5], and advanced computing [6]. With its potential for data storage estimated as being up to 1 Exabyte/mm^3^ and a half-life of over 500 years [7], DNA is a promising candidate for digital information storage, which is becoming particularly essential given the global data volume, which is estimated by the International Data Corporation to be at 175 zettabytes by the end of 2025 [8]. The elongated structure of DNA (with a diameter of 2 nm and a typical length of tens of micrometres) promotes the strand-like clustering of doped materials [9,10], which can behave like metallic nanowires. Specifically prepared DNA can also spontaneously assemble in origami structures [11] and other matrices [12].

In contrast to natural DNA, the DNA–cationic surfactant complex is insoluble in water but soluble in alcohols and nonpolar liquids, which facilitates the production of thin layers and significantly reduces the influence of humidity on its properties. Thus, it is particularly important in electronics applications [13,14]. In addition, the good thermal stability of such complexes [15] and excellent optical transparency in a wide range of the spectrum, including the visible part [5,12], render them promising materials for advancing molecular optical and optoelectronic fields [15,16]. Such complexes were recently used as, for example, matrices for doping agents exhibiting nonlinear optical properties [12], which are sought after in photonics applications. Other research has shown that DNA–cationic surfactant structures, embedded with organic light-emitting materials and gold nanorods, offer potential applications as filters, blocking layers, and sensors [17] in various industries. Another study indicated that devices utilising DNA–cationic surfactant complex (CTMA) layers could emulate the memory and learning behaviours of biological synapses, including synaptic plasticity, with excellent endurance over intensively repeated stimulation, thus opening the way for neuromorphic computer technology [18].

The biophysical properties of bulk DNA solutions are well studied and well understood; however, there is still a need to investigate the properties, observe phenomena, and study the behaviours of DNA complexes in both bulk and thin layer forms. DNA–cationic surfactant complexes are often investigated as thin layers on various substrates, usually produced by spin-casting or evaporation of the alcohol solvent [5,19], as well as ultrathin layers obtained by molecular beam sputtering [20]. The regular use of thin layers in organic electronics requires precise control of the morphology and thickness of the produced thin layer, its stability under monitored conditions, and easy and economical methods of film production and deposition.

Addressing this idea, we used a two-step technique combining a Langmuir monolayer’s formation on a free water surface (Langmuir–Blodgett scheme) and its vertical transfer onto a solid substrate. This method was successfully utilised to obtain linear self-assembled patterns of the first DNA-CTMA complex [21]. In this study, we extended the technique to three lipoplexes formed as DNA–cationic surfactant complexes, i.e., DNA complexes with cetyltrimethylammonium chloride (CTMA), benzyldimethylhexadecylammonium chloride (BAC), and hexadecylpyridinium chloride (HDP) solutions deposited on mica solid plates. These three surfactants were specifically selected for their aliphatic chain morphology, each containing 16 carbon atoms, to investigate the influence of their different headgroups on the formation of ordered structures in thin films. It has been argued in various studies that the length of the aliphatic chains determines the formation of complexes. Chains with more than 16 carbon atoms are usually water-insoluble, thus hindering complex formation. On the other hand, chains with fewer than 16 carbon atoms cannot secure the adequate mechanical properties of the complexes. Physiochemical characterisation of powdered DNA complexes based on plasmid and linear DNA with the selected surfactants was reported elsewhere [22,23].

In this study, six different complexes were synthesised and investigated, combining one of two DNA types: plasmid DNA (pDNA) and low-molecular-weight, linear, double-stranded DNA (dsDNA) with one of three cationic surfactants: (I) CTMA, (II) BAC, or (III) HDP. The formation of stabilised, linear, self-assembled patterns in thin films of DNA–cationic surfactant complexes deposited on a mica substrate was compared at different constant surface pressures during compression on a water subphase to optimise the deposition conditions.

## 2. Materials and Methods

### 2.1. Materials

Plasmid DNA, phMGFP (Promega, Madison, WI, USA), was extracted from *E. coli* bacteria harvested in a laboratory with the NucleoBond PC 10000 EF Giga kit for endotoxin-free plasmid DNA (Macherey-Nagel GmbH & Co. KG, Düren, Germany) according to the delivered procedure, as described in [24]. Low-molecular-weight DNA (dsDNA); cationic surfactants CTMA (CH_3_(CH_2_)_15_N(Cl)(CH_3_)_3_), HDP (C_21_H_38_ClN·H_2_O), and BAC (CH_3_(CH_2_)_15_N(Cl)(CH_3_)_2_CH_2_C_6_H_5_); Tris base (tris(hydroxymethyl) aminomethane); and hydrochloric acid (HCl) were used as received from SIGMA Aldrich (St. Louis, MO, USA). Detailed structural formulas of CTMA, HDP, and BAC surfactants were published previously [22] and are therefore not reproduced here.

### 2.2. Methods

The surface pressure–area (π-A) isotherms were acquired using a Langmuir trough (NIMA) equipped with two movable barriers, providing a total working area of 503 cm^2^. Surface pressure was measured with a precision of ± 0.1 mN/m using a Wilhelmy plate fashioned from ashless chromatography paper (Whatman). Before each recording, the aqueous subphase was cleaned by fully closing the barriers and aspirating the surface layer until pressure fluctuations remained within ± 0.1 mN/m, in agreement with those taken with open barriers. Temperature control of the subphase was achieved through a thermostated circulation system (Julabo GmbH, Seelbach, Germany), maintaining a stable 20 °C ± 0.1 °C environment. The spreading solution was applied to the air–water interface using a 250 μL Hamilton microsyringe, ensuring droplet precision at 5.0 μL. Following application, the monolayer was allowed a 5 min equilibration period before compression at a rate of 20 cm^2^/min. Each π-A isotherm measurement was performed in at least duplicate to verify reproducibility.

The monolayer structure was further analysed using a Brewster angle microscope (BAM, Accurion GmbH, Göttingen, Germany) to support interpreting experimental outcomes. The setup employed a 50 mW laser emitting p-polarised light at 658 nm and was coupled with a 10× magnifying objective. The BAM was mounted above a KSV NIMA Langmuir trough (KSV Instruments Ltd., Espoo, Finland) featuring dual barriers and offering a surface area of 841 cm^2^.

The DNA–surfactant films were compressed to specific surface pressures (5, 10, 15, and 20 mN/m), which were kept constant for further analysis. Surface pressures above 20 mN/m were not pursued because all complexes collapsed at π_coll_ ≈ 22–29 mN/m (Table 1); compression beyond this threshold produced film break-up in BAM and discontinuous, aggregated deposits, precluding reliable Langmuir–Blodgett transfer. Concurrently, changes in the monolayer area were tracked over time. Langmuir monolayers were deposited onto mica substrates using the Langmuir–Blodgett (LB) transfer method. Before each deposition, the solid substrate was immersed in the aqueous subphase. Once the complex solution had been spread over the water surface, the film was allowed to equilibrate for 5 min, after which it was compressed to a predefined surface pressure of 5, 10, 15, or 20 mN/m. The transfer process was performed by vertically withdrawing the substrate through the monolayer at a 5 mm/min controlled speed using a mechanical dipper. The substrates used were V1-grade ruby muscovite mica sheets sourced from Continental Trade (Continental Trade Sp. z o.o., Nadma, Poland). Immediately before each deposition, the mica surfaces were cleaned using adhesive tape to remove surface impurities.

Because the DNA strands were charge-neutralised by cationic surfactants before spreading, the resulting hydrophobic complexes adhered uniformly to freshly cleaved mica; preliminary trials showed no need for additional polylysine or Mg^2+^ pretreatment, which can over-adsorb soft organic layers and alter their morphology.

AFM images were procured using non-contact mode. This was performed in ambient conditions employing an Agilent 5500 Microscope (Agilent Technologies, Inc., Santa Clara, CA, USA). The resonance frequency stayed between 45 and 115 Hz. The tips adopted were from a nanosensor of the PPP-FMR-50 type with a 3.0(1) µm thickness. Topography images were acquired in various locations on each sample, with 512 × 512 lines. During the measurement, gains and set-points were regulated to maximise clarity and minimise noise for each scan. The measuring speed was set independently, ranging from 0.9 to 1.3 lines/s. Scan results were handled by Gwyddion 2.61 software. Resulting images were 2.5 µm × 2.5 µm, chosen as representative for every DNA type complex. After thresholding with Otsu’s algorithm, the coverage percentage (P) and mean volume of the structures (V) were calculated. The coverage percentage was obtained by using the projected area function of the part of the images marked above the threshold. The mean height value was calculated by subtracting the mean height of the background from the mean height of structures marked as above the threshold. The mean volume of the domains was calculated by multiplying these two values. It could be interpreted as the volume of material deposited on the substrate in the area that had been imaged. Depth profiles provided by AFM were analysed using the fast Fourier transform (FFT) algorithm embedded in the OriginPRO 2019 software package.

## 3. Results and Discussion

### 3.1. Langmuir Monolayer Technique

**Figure 1:**
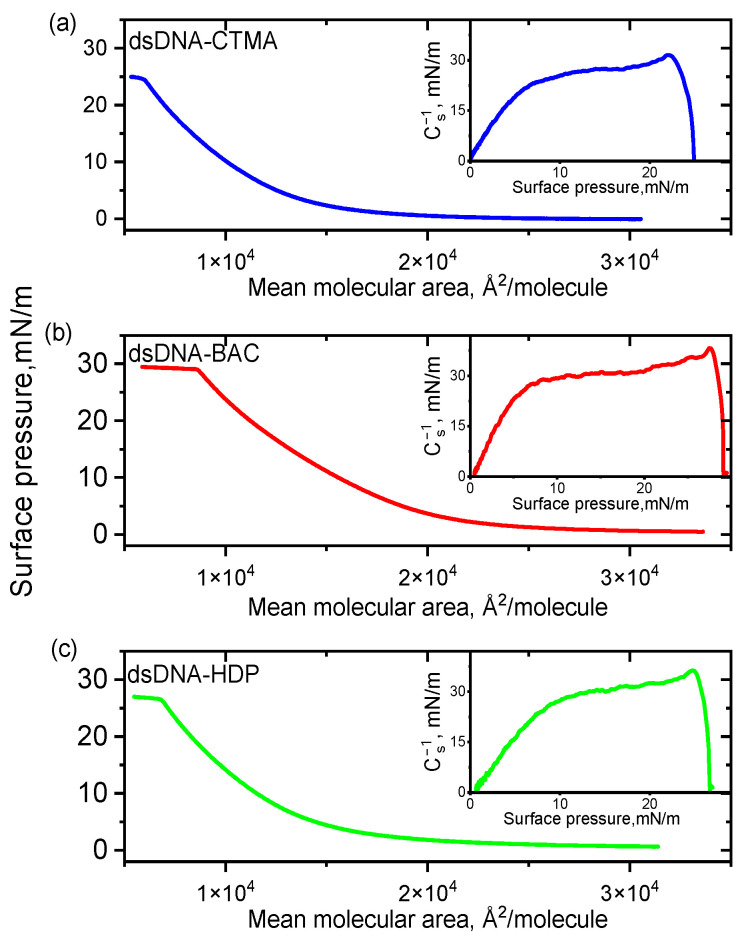
π–A isotherms for dsDNA complexes with cationic surfactants CTMA (**a**), BAC (**b**), and HDP (**c**) spread on water subphase at 20 °C. Insets: compression moduli as a function of surface pressure (π).

**Figure 2:**
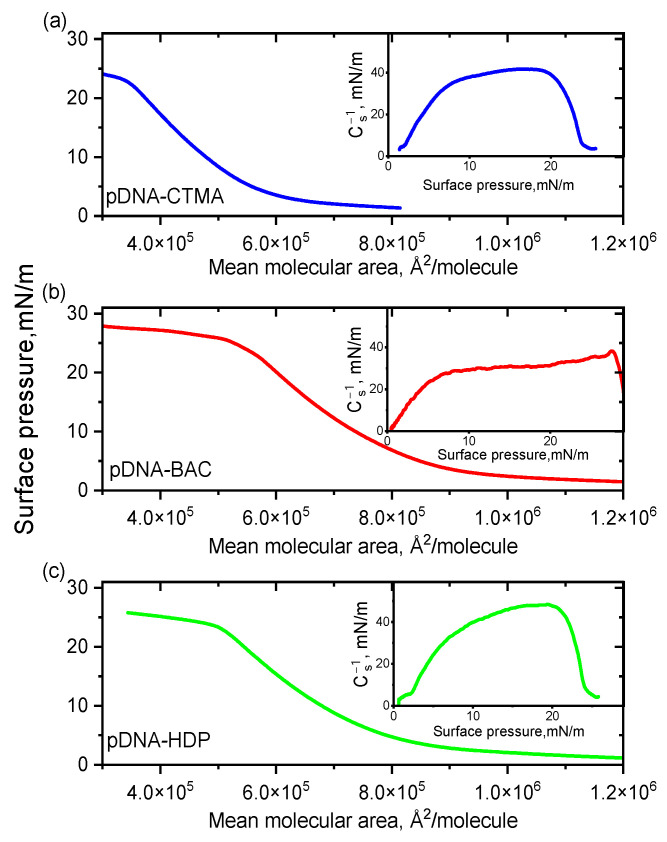
π–A isotherms for pDNA complexes with cationic surfactants CTMA (**a**), BAC (**b**), and HDP (**c**) spread on water subphase at 20 °C. Insets: compression moduli as a function of surface pressure (π).

Langmuir balance studies were carried out to investigate the monolayer behaviour of complexes based on two DNA architectures (linear dsDNA and plasmid pDNA) in combination with three distinct surfactants. Surface pressure-area (π-A) isotherms and corresponding compression moduli versus the surface pressure registered for complexes based on linear and plasmid DNA are presented in Figure 1 and Figure 2, respectively. All recorded isotherms exhibited similar profiles, and an analysis of the results indicated that the surfactant structure had no significant influence on the shape and position of the DNA complexes’ isotherms, regardless of DNA type.

The collapse pressure and compressibility coefficient for all complexes synthesised are presented in Table 1. The highest value of the collapse pressure was recorded among all linear DNAs studied for the dsDNA-BAC complex, while for the dsDNA-CTMA complex, it was the lowest. In turn, for the pDNA-CTMA complex, an isotherm rise (surface pressure increase) was observed for a surface of about 600 kÅ^2^/molecule, i.e., for a smaller surface than in the cases of the pDNA-BAC and pDNA-HDP complexes (about 800 kÅ^2^/molecule), while the collapse pressures of the monolayers were similar (Table 1). The determined compressibility coefficients indicated that the monolayers for the pDNA-CTMA and pDNA-HDP complexes were in an expanded liquid state, while pDNA-BAC was in an intermediate state between an expanded liquid and a condensed liquid. On the other hand, all complexes based on linear DNA collapsed in the expanded liquid state. All compressibility coefficients for the pDNA-based complexes were clearly higher than the analogous coefficients for the linear DNA-based complexes. These differences probably resulted from the three spatial conformations in which the plasmid DNA occurred (circular, linear, and supercoiled, Ref. [24]).

**Figure 3:**
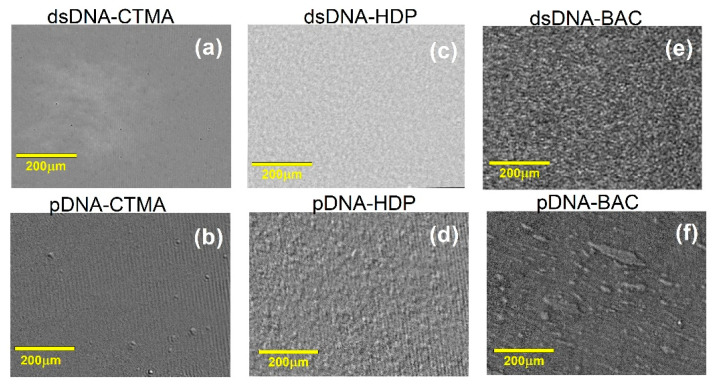
BAM images recorded for dsDNA and pDNA complexes with cationic surfactants CTMA (**a**,**b**), BAC (**c**,**d**), and HDP (**e**,**f**) at collapse pressure π_coll_.

Figure 3 presents BAM images (a–f) acquired at the respective collapse pressures (*π_coll_*) for the dsDNA- and pDNA-based complexes. Only the pDNA–BAC monolayer displayed bright, condensed domains, whereas the remaining films (e.g., pDNA–CTMA and all dsDNA complexes) stayed uniformly dark, indicating an expanded state at collapse. These visual contrasts corroborate the isotherm and compressibility data: plasmid DNA films began domain condensation at lower surface pressures, while linear DNA complexes remained expanded until higher pressures. Thus, Figure 3 offers direct morphological evidence that DNA architecture governs monolayer behaviour under extreme compression.

### 3.2. AFM Imaging and FFT Analysis

**Figure 4:**
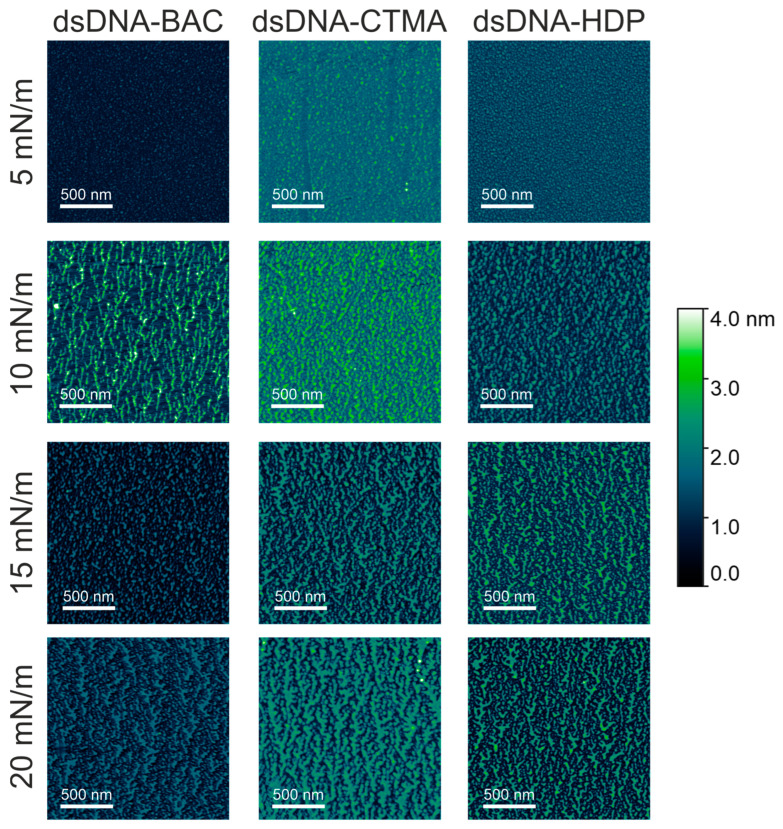
AFM images of dsDNA–based complexes deposited on mica slides obtained from monolayers transferred at different surface pressures. The effect of increasing compression pressure can be observed from **top** to **bottom**.

**Figure 5:**
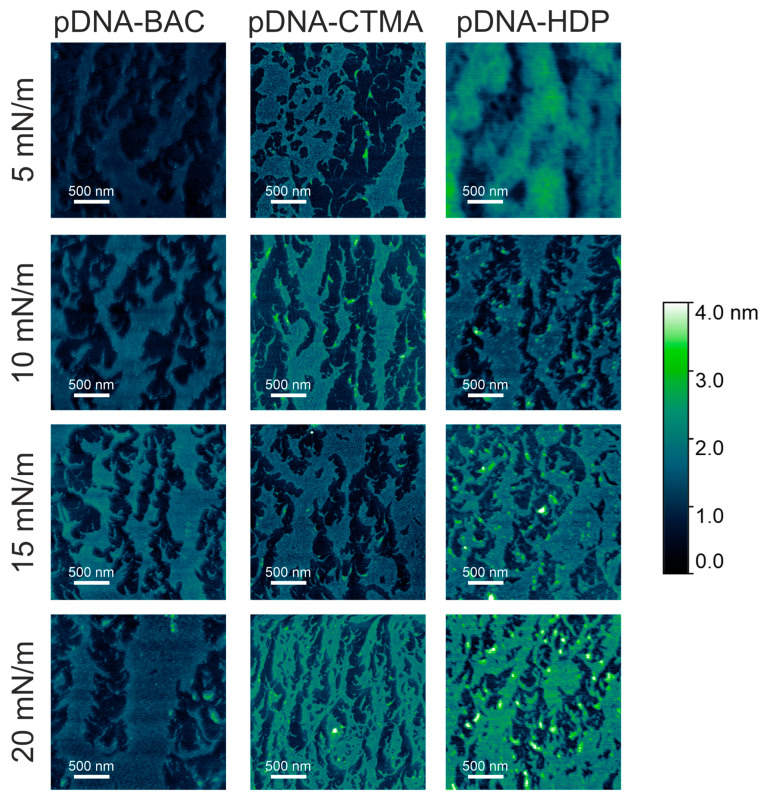
AFM images of plasmid pDNA–based complexes deposited on mica slides obtained from monolayers transferred at different surface pressures. The effect of increasing compression pressure can be observed from **top** to **bottom**.

All the complexes were deposited on a solid support (mica) at surface pressures of 5 mN/m, 10 mN/m, 15 mN/m, and 20 mN/m and imaged by atomic force microscopy. AFM images registered for linear and plasmid DNA complexes at the chosen surface pressure are presented in Figure 4 and Figure 5, respectively. AFM analysis revealed that the narrowest stripes obtained for dsDNA–CTMA at 15 mN/m were only 21 nm wide, confirming that the lateral resolution of the method reached the low tens-of-nanometres range.

In the case of dsDNA-BAC layers, the spontaneous formation of elongated structures was observed for samples lifted at surface pressures of 10 and 20 mN/m, while their absence was observed for surface pressures of 5 and 15 mN/m. The presence of structures (for pressures of 10 and 20 mN/m) and some periodicity in the direction perpendicular to the direction of sample lifting are well visible in Figure 4. In the case of the dsDNA-HDP complex, the successive formation of elongated structures was observed with increasing surface pressure, similar to the dsDNA-CTMA complex. No disturbance of this trend was observed for the surface pressure of 15 mN/m as it was for dsDNA-BAC (Figure 4).

**Figure 6:**
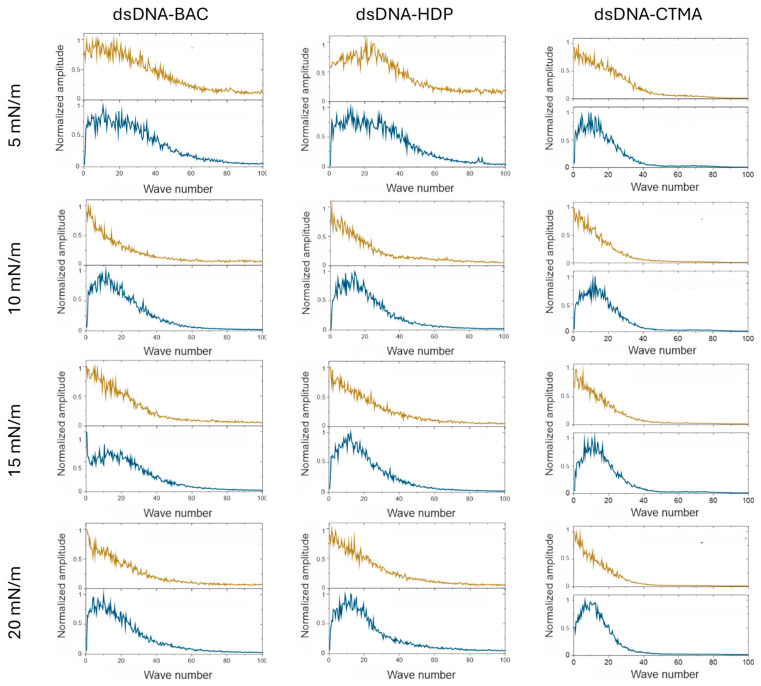
Results of the FFT analysis performed on the depth profile data extracted from AFM images. Graphs are organised in the same way as images in Figure 4. Yellow lines are results for the direction parallel to the structures and blue lines are results for the direction perpendicular to the structures.

**Figure 7:**
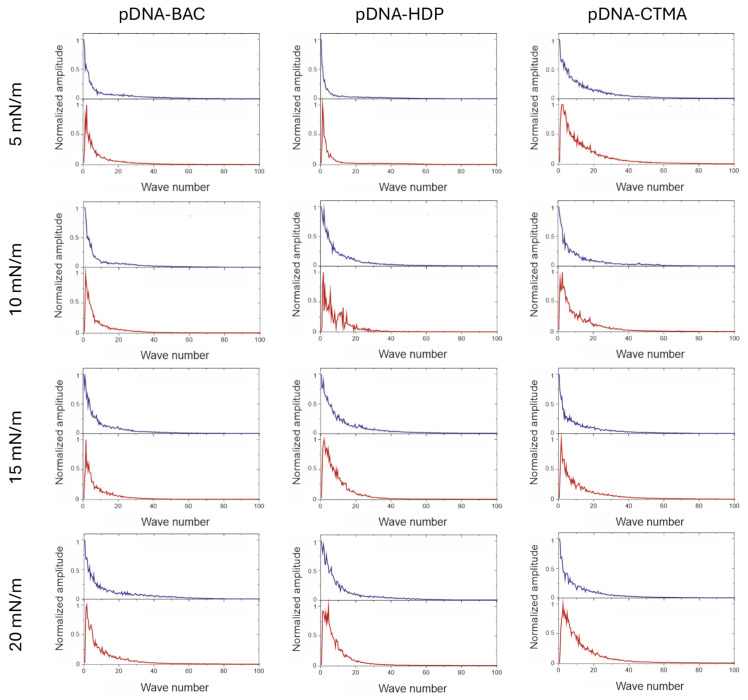
Results of the FFT analysis performed on the depth profile data extracted from AFM images. Graphs are organised in the same way as images in Figure 5. Blue lines are results for the direction parallel to the structures and red lines are results for the direction perpendicular to the structures.

**Figure A1:**
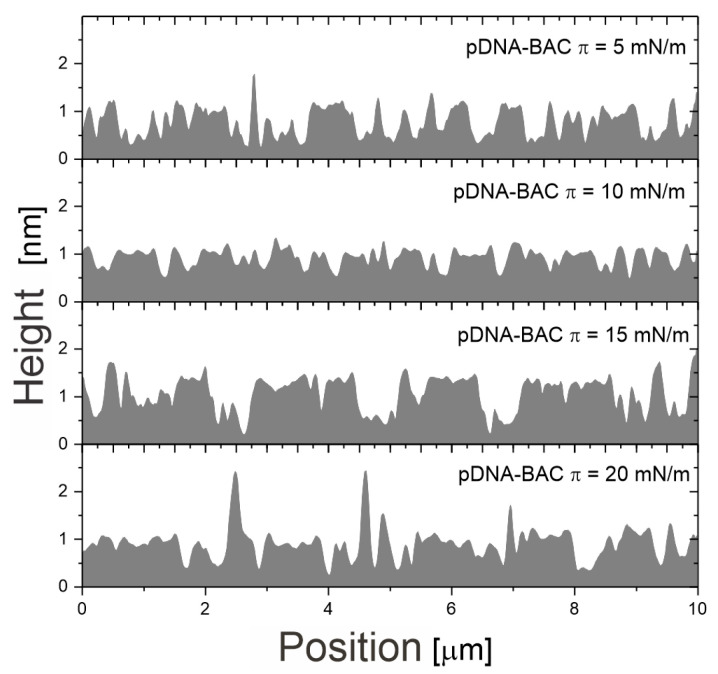
Representative example of depth profiles of AFM images obtained for pDNA-BAC layers.

Fast Fourier transform analysis was performed on depth profile data extracted from AFM images (Figure A1). FFT analysis of AFM images in a direction parallel to the sample extraction direction resulted in a monotonic decrease in value, indicating a lack of periodicity. In the graphs of profiles perpendicular to the sample lifting direction, a sharp maximum was observed in the image analysis, which indicated periodicity (see Figure 6 and Figure 7). For all recorded AFM images, complementary FFT analysis was performed, and the occurrence of periodic structures was confirmed for profiles perpendicular to the sample lifting direction, as well as their absence in the parallel direction for all complexes based on plasmid pDNA and part of the complexes based on linear dsDNA.

The average width of the structures was determined by analysing the height profiles of the complexes; the results are gathered in Table 2. In the case of linear dsDNA complexes and a surface pressure of 20 mN/m, it was found that for the dsDNA-CTMA complex, the width of elongated structures (stripes) was ca. 37 nm, while for the complexes with the other two surfactants (BAC and HDP), it was ca. 25 nm. In turn, for pDNA-based complexes, the average widths determined at the same surface pressure (20 mN/m) based on height profiles (Figure 7) were 122(47) nm, 159(70) nm, and 195(71) nm for the pDNA-CTMA, pDNA-HDP, and pDNA-BAC complexes, respectively. The average structure widths for each surface pressure are gathered in Table 2. For the pDNA-BAC complex layers, an increase in the obtained structures was observed with increasing surface pressure. In turn, the pDNA-CTMA and pDNA-HDP complex layers at the pressure of 20 mN/m seemed much more tightly compressed, making the structures appear more tightly packed. Despite the more compressed layer, the width of the observed structures was not significantly smaller than for the layers obtained at a lower surface pressure.

The main difference observed between the layers formed by plasmid pDNA-based complexes (Figure 5 and Figure 7) and linear dsDNA-based complexes (Figure 3 and Figure 5) was the thickness of the stripes and the minimum surface pressures at which elongated structures were formed. For complexes based on plasmid pDNA, structures were observed in each case from the lowest surface pressure of 5 mN/m. An apparent increase in the width of the structure with increasing surface pressure was observed for the pDNA-BAC complex. For the other layers studied, the width of the obtained shapes was similar (see Table 2). FFT analysis confirmed the lack of periodicity in the direction parallel to the direction of lifting the sample, while, perpendicular to this direction, it showed the occurrence of function maxima, allowing for the determination of distances between structures. In the case of the pDNA-BAC complex, for a surface pressure of 20 mN/m, very-well-separated structures were observed (the distance between them was determined to be about 0.78 μm), and more packed structures for the pDNA-CTMA and pDNA-HDP complexes were found (distances of about 0.4 μm and 0.6 μm, respectively). These distances correlated well with the visual analysis of AFM images—the pDNA-CTMA layer with a surface pressure of 20 mN/m seemed to be the most compressed.

**Figure 8:**
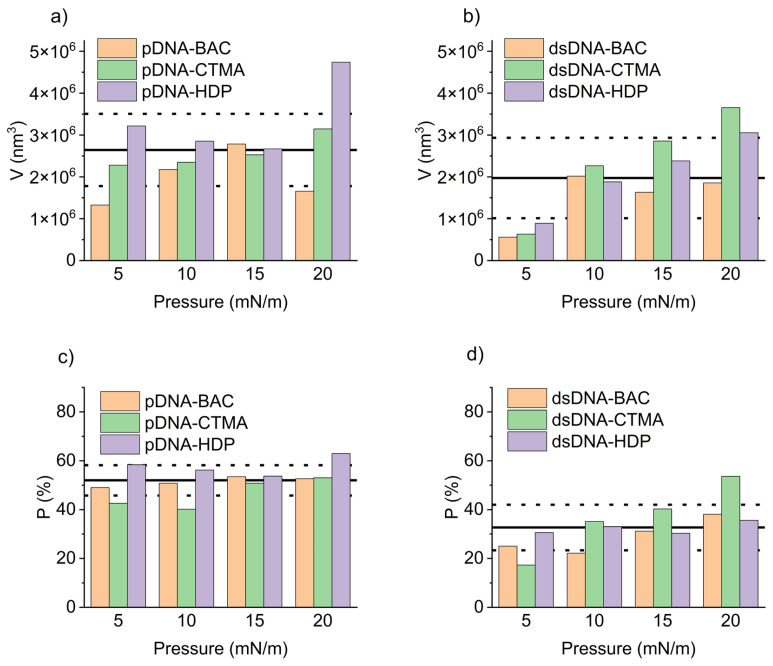
Mean volume of the domains for pDNA-based complexes (**a**) and dsDNA-based complexes (**b**) along with the percentage of surface coverage for pDNA-based complexes (**c**) and dsDNA-based complexes (**d**) as obtained from representative AFM images. Solid lines indicate the mean values, dashed lines—standard deviation.

The mean volume and percentage of surface coverage obtained from representative AFM images of plasmid pDNA-based and linear dsDNA-based complexes are presented in Figure 8a,c and Figure 8b,d, respectively. For all pDNA-based complexes, the mean volume of surface structures remained largely unaffected by increasing deposition pressure (Figure 8a). In contrast, their surface coverage was essentially constant across the tested pressures, with an average value of 52.0 ± 6.2% (Figure 8c). For the remaining graphs, the average value of the presented parameters, along with the standard deviation, is also presented to highlight observed trends. This behaviour was likely related to the high compressibility coefficient of the pDNA-based films during deposition (Table 1), producing stiffer and more densely packed layers that limited structural rearrangement under vertical transfer. Linear dsDNA-based complexes, characterised by lower compressibility coefficients (Table 1), exhibited an apparent increase in the mean volume of surface structures with increasing deposition pressure (Figure 8b). This pressure-dependent trend was evident for both dsDNA-CTMA and dsDNA-HDP complexes and, apart from a slight deviation at 10 mN/m, also for dsDNA-BAC. Concerning the surface coverage (Figure 8d), the dsDNA-CTMA and dsDNA-HDP complexes showed a monotonic increase with pressure. In contrast, the dsDNA-BAC sample deposited at 5 mN/m displayed markedly lower coverage, most likely because insufficient compression limited effective molecular organisation and self-assembly. Because stripe emergence was pressure-dependent (e.g., dsDNA-BAC displayed patterns of only ≥10 mN/m), surface-pressure programming or sequential masked transfers could be used to print well-defined, microarray-like DNA patterns. Recent studies demonstrated that reactive polyamines can trigger the controlled folding and compaction of DNA nanostructures [25], a prerequisite for their use as programmable drug-delivery vehicles [26]. The cationic surfactants employed here fulfilled a similar charge-neutralising role, suggesting that our folded DNA films constitute a viable foundational platform for future therapeutic cargo loading. Attempts to deposit unmodified pDNA and dsDNA under identical Langmuir–Blodgett conditions resulted only in isolated, randomly adsorbed filaments with no long-range order, in agreement with earlier reports [27,28]; thus, the periodic stripe patterns shown in Figure 4 and Figure 5 arose solely from cationic surfactant-driven condensation rather than from native DNA itself.

## 4. Conclusions

Monolayer behaviour at the air–water interface was studied, the protocol for their deposition onto mica substrates was refined, and the resulting structures were characterised via atomic force microscopy. The registered AFM images were analysed using the fast Fourier transformation (FFT) method. Two types of DNA (linear dsDNA and plasmid pDNA) were used to synthesise complexes with three cationic surfactants (CTMA, BAC, HDP). Thin layers of each complex were deposited onto mica slices using the Langmuir–Blodgett (LB) technique. The obtained results demonstrated that the LB method enabled the formation of monolayers with distinct structures depending on the lifting conditions, DNA type, and surfactant type. The main difference between the layers formed by pDNA-based and dsDNA-based complexes was the thickness of the stripes and the minimum surface pressures at which elongated structures (stripes) were observed. In pDNA-based complexes, these structures appeared starting from the lowest tested surface pressure of 5 mN/m, whereas dsDNA-based complexes were only observed at the highest surface pressure of 20 mN/m. The conducted studies allowed the following general conclusions to be drawn:

- DNA–cationic surfactant complexes form monolayers at the liquid–gas interface, which spontaneously self-organise during transfer onto the mica surface under specific boundary conditions (surface pressure 10–20 mN/m, lifting speed 5 mm/min).
- FFT analysis of AFM images confirmed the formation of elongated structures aligned with the lifting direction.
- The type of DNA (linear or plasmid) mainly influences the appearance of surface structures, while the surfactant structure has a much smaller effect.
- The type of DNA does not significantly impact the shape of the isotherm, including the monolayer collapse pressure.
- The type of DNA influences the percentage of surface coverage; for plasmid pDNA-based complexes, the coverage remains nearly constant at approximately 52%, higher than that observed for linear dsDNA-based complexes.

Moreover, this study demonstrated that the Langmuir–Blodgett technique enables precise control over the structure of thin films of DNA–cationic surfactant complexes. It was shown that the type of DNA (linear dsDNA versus plasmid pDNA) significantly affected the morphology of the formed layers, whereas the surfactant structure played a relatively minor role. Plasmid DNA-based complexes formed elongated structures at lower surface pressure and exhibited broader stripe widths than linear DNA-based complexes. FFT analysis confirmed the presence of the structures visible on AFM images. Our results highlight the potential of DNA-based complexes in organic electronic applications, although further optimisation is required to enable studies of the electrical properties. In summary, the properties of thin layers of DNA–cationic surfactant complexes depend mainly on the type of DNA, with no apparent influence of the surfactant structure observed. However, the impact of the surfactant type and DNA structure on the electrical properties of these layers cannot be entirely ruled out. Unfortunately, the layers produced were too thin at the current research stage to allow conductivity measurements. Therefore, further optimisation of the production method and a more detailed investigation of the properties of DNA–cationic surfactant layers are necessary. Importantly, spectroscopic and X-ray characterisation of the same DNA–surfactant complexes demonstrate retention of the A-form double helix; together with the reproducible formation of micrometre-long stripes, this indicates that DNA remains structurally intact and, after gentle rehydration or surfactant exchange, should remain capable of hybridisation or particle binding.

## Figures and Tables

**Table 1:** The collapse pressure π_coll_ and compressibility coefficient C_s_^−1^ for all complexes studied.

	π_coll_ [mN/m]	C_s_^−1^ [mN/m]
pDNA-CTMA	22(0.4)	42(1)
pDNA-BAC	25(0.7)	52(1)
pDNA-HDP	23(0.7)	48(2)
dsDNA-CTMA	25(0.2)	30(1)
dsDNA-BAC	29(0.5)	36(2)
dsDNA-HDP	26(0.5)	35(1)

**Table 2:** Mean structure width obtained by analysing the height profiles of the complexes studied.

Surface Pressure [mN/m]	pDNA-CTMA [nm]	pDNA-BAC [nm]	pDNA-HDP [nm]	dsDNA-CTMA [nm]	dsDNA-BAC [nm]	dsDNA-HDP [nm]
5	120(49)	134(57)	150(63)	-	-	-
10	114(50)	178(76)	160(68)	-	-	-
15	115(44)	182(85)	162(65)	21(9)	-	-
20	122(47)	195(71)	159(70)	38(12)	23(10)	26(10)

## Data Availability

The original contributions presented in this study are included in the article. Further inquiries can be directed to the corresponding author.
